# Weather constraints on global drone flyability

**DOI:** 10.1038/s41598-021-91325-w

**Published:** 2021-06-08

**Authors:** Mozhou Gao, Chris H. Hugenholtz, Thomas A. Fox, Maja Kucharczyk, Thomas E. Barchyn, Paul R. Nesbit

**Affiliations:** 1 Department of Geography, University of Calgary, 2500 University Drive NW, Calgary, AB T2N 1N4 Canada; 2 Department of Geoscience, University of Calgary, 2500 University Drive NW, Calgary, AB T2N 1N4 Canada

**Keywords:** Climate sciences, Atmospheric science, Atmospheric dynamics

## Abstract

Small aerial drones are used in a growing number of commercial applications. However, drones cannot fly in all weather, which impacts their reliability for time-sensitive operations. The magnitude and global variability of weather impact is poorly understood. We explore weather-limited drone flyability (the proportion of time drones can fly safely) by comparing historical wind speed, temperature, and precipitation data to manufacturer-reported thresholds of common commercial and weather-resistant drones with a computer simulation. We show that global flyability is highest in warm and dry continental regions and lowest over oceans and at high latitudes. Median global flyability for common drones is low: 5.7 h/day or 2.0 h/day if restricted to daylight hours. Weather-resistant drones have higher flyability (20.4 and 12.3 h/day, respectively). While these estimates do not consider all weather conditions, results suggest that improvements to weather resistance can increase flyability. An inverse analysis for major population centres shows the largest flyability gains for common drones can be achieved by increasing maximum wind speed and precipitation thresholds from 10 to 15 m/s and 0–1 mm/h, respectively.

## Introduction

Small (< 25 kg) uncrewed aircraft systems (sUAS), or drones, are an emerging technology with considerable speculation surrounding their potential to disrupt a wide range of civilian and commercial sectors^1–3^. Before 2014, investment in drones focused primarily on meeting government, military, and surveillance needs^1^. By 2017, recreational and commercial drones were a billion-dollar industry in the US and projected to quadruple to $11.8 billion by 2026^4^. In September 2020, there were > 1.7 million drones registered with the United States Federal Aviation Authority (FAA), 30% of which were registered for commercial applications^5^. As existing applications mature, new uses are being tested that may transform commercial sectors, with drones supplanting conventional methods and adding new services^3,6^. Growth in drone utilization has been accompanied by rapidly evolving legislation and initiatives to modernize airspace for the safe integration of drones^7,8^. However, the transition towards widespread and on-demand drone applications and services requires consideration of a wide variety of factors^9^.

Weather is an important and poorly resolved factor that may affect ambitions to expand drone operations^10^. The drone industry lags behind the aerospace industry in the development and implementation of standards for weather-related testing and tolerances^11^. In an exercise to outline the status of drone technical and performance standards, the American National Standards Institute (ANSI) identified weather robustness as an important gap and high priority^12^. Few published standards or specifications exist specifically targeting weather effects on drone flight performance and safety^12^.

Air temperature, wind speed, precipitation, and other atmospheric phenomena have been shown to adversely affect drone endurance, control, aerodynamics, airframe integrity, line-of-sight visibility, airspace monitoring, and sensors for navigation and collision avoidance^10,11^. There are situations when most drones should not and cannot fly (i.e., ‘severe’ weather hazards as categorized by Ranquist et al.^10^)—but understanding where, when, and how adverse and severe weather conditions arise and impact drone operations is complicated. Researchers have documented the current weather resources and tools available to assess weather-related risks for local drone operations^11,13–15^. Roseman and Argrow^11^ created a risk-based framework to assess safe operations based on weather forecast, population density, structure density, and drone specifications. Lundby et al.^14^ compared historical weather data and four drone platforms to determine the percentage of the year when drone operations were possible in two different cities. Their results demonstrate that local weather, relative to specific drone tolerances, can substantially impact drone operations and limit flights to between 53.9 and 95.8% of the year^14^. Risk-based frameworks and high-resolution datasets are imperative for assessing safe operations locally, but the extent to which different drone types are limited by weather in different parts of the world remains unknown.

Many drone manufacturers specify safe operating limits or warnings according to weather parameters in their manuals (e.g., “Do not use the aircraft in severe weather conditions including wind speeds exceeding 10 m/s, snow, rain, and fog”^16^). Regulations in some jurisdictions specify that drone operations cannot proceed unless weather conditions at the time of flight permit operation in accordance with the manufacturer’s instructions^17^. To comply with drone legislation, drone operators must maintain flexible deployment schedules to allow for delays or cancellations due to weather conditions outside the manufacturer-specified operating envelope^18^. However, to achieve wider adoption of drones for time-sensitive operations such as emergency response, law enforcement, and package delivery, drones will be more effective if they are not limited by weather. The value of using drones for these applications is diminished if the drones cannot perform with reasonable uptime.

Technical and legal limitations to drone flights have material consequences for the economics, utility, and reliability of drones for on-demand commercial operations—particularly if the application has pre-existing service targets. For example, even if package delivery with drones is preferable, delivery companies require on-demand capacity to meet delivery targets on the days when drones cannot fly. Similarly, the use of drones for emergency response or law enforcement is attractive, but a full complement of back-up tools is required if the drones cannot fly due to inclement weather. Are drones a worthwhile investment if uptime is low? Furthermore, unlike hobby or small-scale scientific drone use, these ambitious commercial applications are particularly exposed to the legalities of stretching operations into poor weather. Commercial applications may inherit significant liabilities if flying in conditions outside drone operating envelopes.

While site-specific studies of the impact of weather on drone operations are helpful^11,13–15^, a global analysis of this issue can reveal broader trends. A global analysis addresses three major questions. First, what is the impact of weather on drone uptime globally, with implications for the future trajectory of the global drone industry? Second, what is the most limiting weather, and required changes to operational envelopes that could improve uptime, with implications for design optimization of drones? And third, where are the best opportunities globally for on-demand commercial drone use with respect to weather?

To address these questions, here we compare global, historical weather data over a 10-year period with manufacturer-provided drone weather tolerances for common commercial and weather-resistant drones. We present the proportion of time various drones can fly safely (termed “flyability”), with and without daylight constraints. Within this global dataset, we examine the 100 most populated cities, where some on-demand drone services and applications such as delivery and emergency services may be applied commonly. We extend our analysis by looking at necessary weather tolerances required to substantially increase flyability in drones. These analyses foundation the maturations necessary to enable broadscale on-demand operations and prioritize future work.

## Results

### Global flyability

To estimate the impact of weather variables on drone flyability, we used modeled global estimates of hourly air temperature at 2 m above ground level, wind speed at 100 m altitude (a representative flying altitude for most basic operations), and total precipitation per hour and compared them to weather tolerances of two representative drone classes over a 10-year period. The global weather data were derived from the European Centre for Medium-Range Weather Forecasts ERA5 reanalysis dataset^19,20^. Weather reanalysis combines observations with modeling to produce a spatially and temporally continuous global dataset of weather parameters. The two drone classes were selected from the US FAA’s list of commercial registrations and manufacturer-provided weather tolerance data (updated 05 May 2020). We define a common drone (CD) class based on the median weather tolerance of the fifty most commonly registered drones. The CD class has an operational temperature range of 0 to 40 °C, maximum wind speed resistance of 10 m/s, and is not suitable to fly in the rain (precipitation threshold of 0 mm/h). This corresponds to the weather tolerance of 52% of active commercial registrations in the list we received from the FAA. We define a weather-resistant drone (WRD) class based on the second most commonly registered small delivery drone, which is the only small delivery drone for which weather tolerance data were provided by the manufacturer. The WRD has an operational temperature range of − 20 to 46 °C, maximum wind speed resistance of 14 m/s, and precipitation tolerance of 50 mm/h.

The CD class represents widely available, commodity drones that are inexpensive to purchase and easy to operate. While WRDs are clearly higher quality, professional grade systems, CDs are very capable and remarkably useful. At present, the commodity availability, high technology, and low capital costs for CDs mean that they are and will continue to be used for research and commercial operations^21^—particularly in lower-income regions of the world.

**Figure 1:**
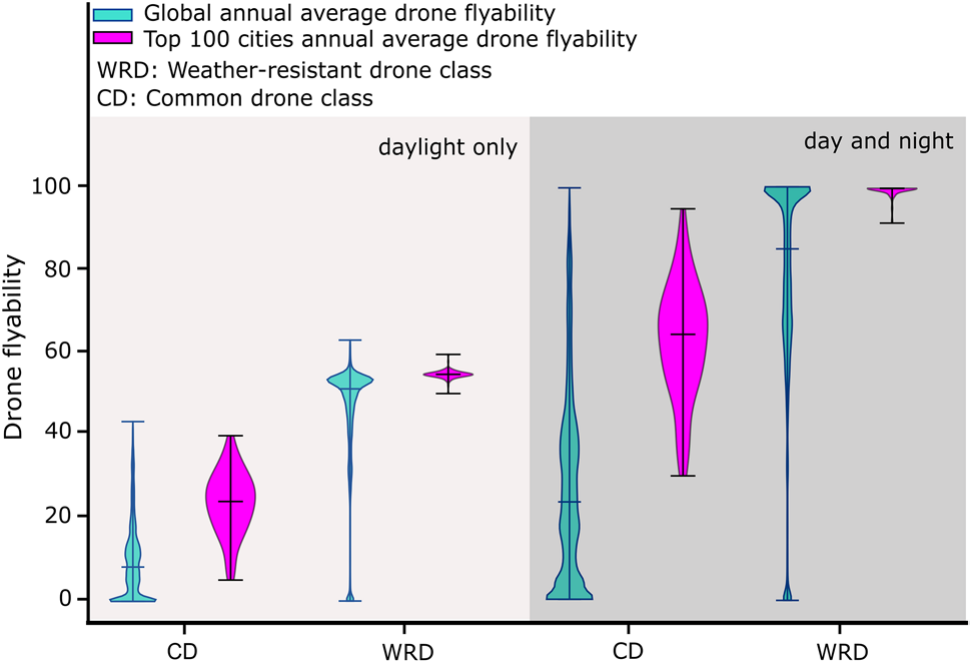
Violin plots of day-and-night and daylight-only flyability for CD and WRD. Cyan violins correspond to the distribution of flyabilities of CD and WRD globally and orange violins represent the distribution of flyabilities of CD and WRD from 100 most populated cities across the world^22^. The upper and lower hinges of violin plots indicate the minimum and maximum flyabilities. The horizontal bar of each violin represents the median of annual average drone flyability.

The 10-year weather analysis reveals that the operational envelopes specified for CDs limit their global (median) day-and-night flyability to 23.6% (5.7 h/day) (Fig. 1). WRDs are capable of much higher global flyability at 85.0% (20.4 h/day). However, these overall flyability percentages do not distinguish between daylight-only and day-and-night operations. Some time-sensitive commercial drone applications, such as business-to-business deliveries or accident scene reconstruction, are typically bracketed by daylight hours. Including daylight-only constraints reduces global flyability to 8.3% and 51.3% for CDs and WRDs, respectively (Fig. 1). Overall, this suggests that typical weather and daylight constraints create barriers to time-sensitive applications using CDs (8.3% flyability equates to 2 h/day of flyable conditions). Moreover, flyability distributions are spatially dependent: for CDs, some regions are flyable nearly year-round, while most regions rarely exhibit suitable operational conditions (or vice versa for WRDs) (Fig. 1).

We examine weather and daylight constraints for the 100 most populated world cities^22^ (Fig. 1). Median day-and-night flyability over these urban areas increases to 64.3% and 99.5% for CDs and WRDs, respectively, and drops to 23.6% and 54.2% in daylight-only operations (Supplementary Data 1). Flyability is extremely variable for CDs, ranging from 94.8% in Alexandria, Egypt, to 29.8% in Singapore. Daylight-only flyability and day-and-night flyability ranges, respectively, from 4.5 to 39.6% and from 29.8 to 94.8% (Supplementary Data 1). These large ranges for CDs demonstrate that weather-constrained flyability varies with geography and that flyability tends to be higher in densely populated regions than on a global basis. Flyability is consistent for WRDs. Daylight-only flyability ranges from 49.5 to 59.0%, and day-and-night flyability ranges from 91.5 to 100.0% (Supplementary Data 1).

### Spatial variability

**Figure 2:**
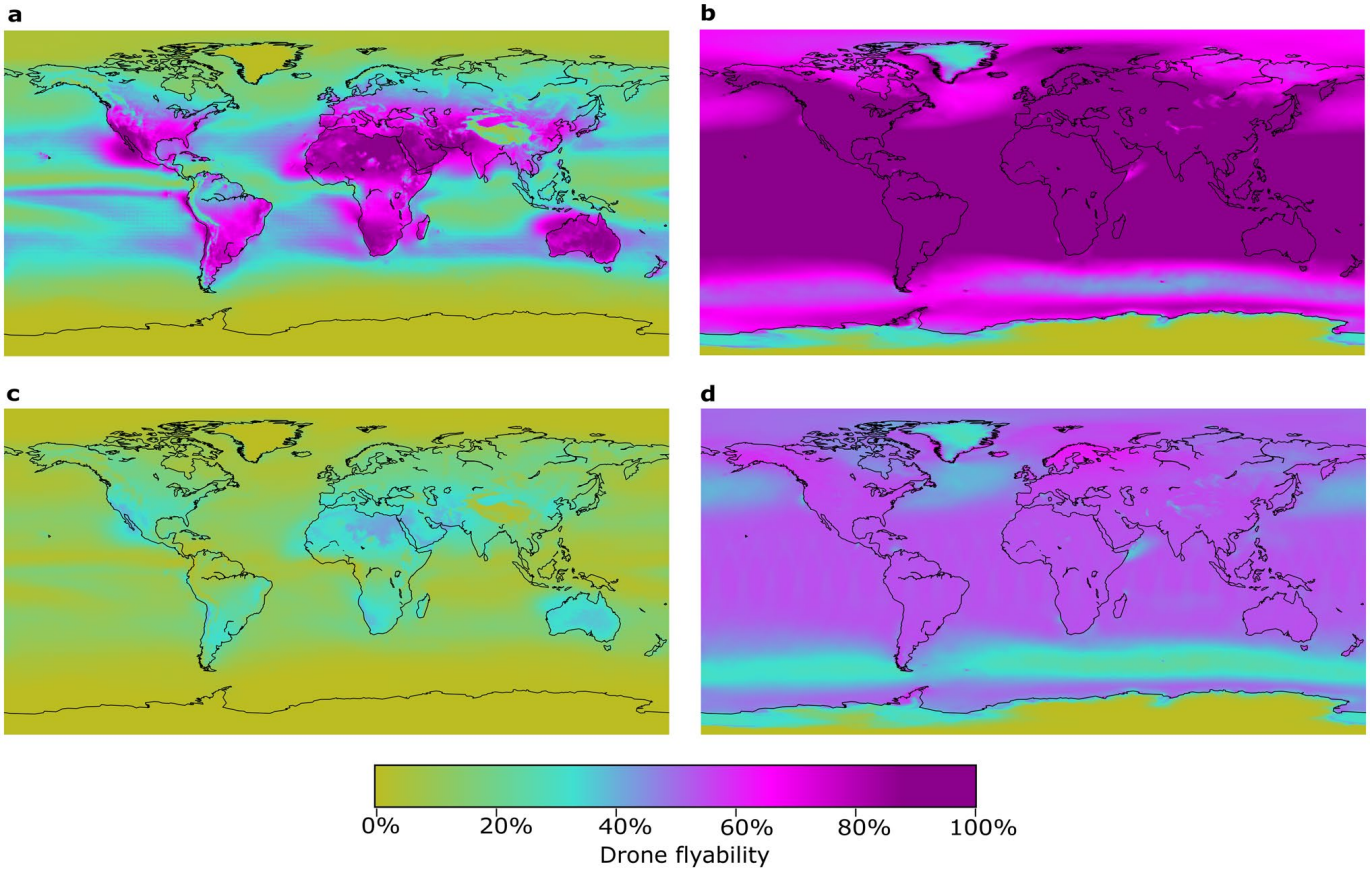
Global flyability: (**a**) CD in day-and-night conditions, (**b**) WRD in day-and-night conditions, (**c**) CD in daytime only conditions, and (**d**) WRD in daytime only conditions.

Flyability varies with climate, generally following a latitudinal gradient in which flyability is lowest in the poles and highest at mid-latitudes and near the equator (Fig. 2). The global pattern outlines favourable and less favourable drone climates. Generally, drones have higher flyability over land than over large bodies of water due to characteristically higher wind speeds and the larger spatial footprint of precipitation. This is particularly apparent for CDs (Fig. 2, left panels), which are more sensitive to precipitation. Regions with the highest drone flyability are found in hyper-arid to sub-humid environments (e.g., northern and southern Africa, southwestern North America, Australia) that have year-round warm temperatures, low to moderate winds, and limited precipitation. High elevation regions associated with prominent mountain ranges (e.g., Rocky Mountains, Andes, and Himalayas) also appear as areas with reduced flyability, particularly for CDs due to lower temperatures, and higher precipitation and/or wind speeds.

**Figure 3:**
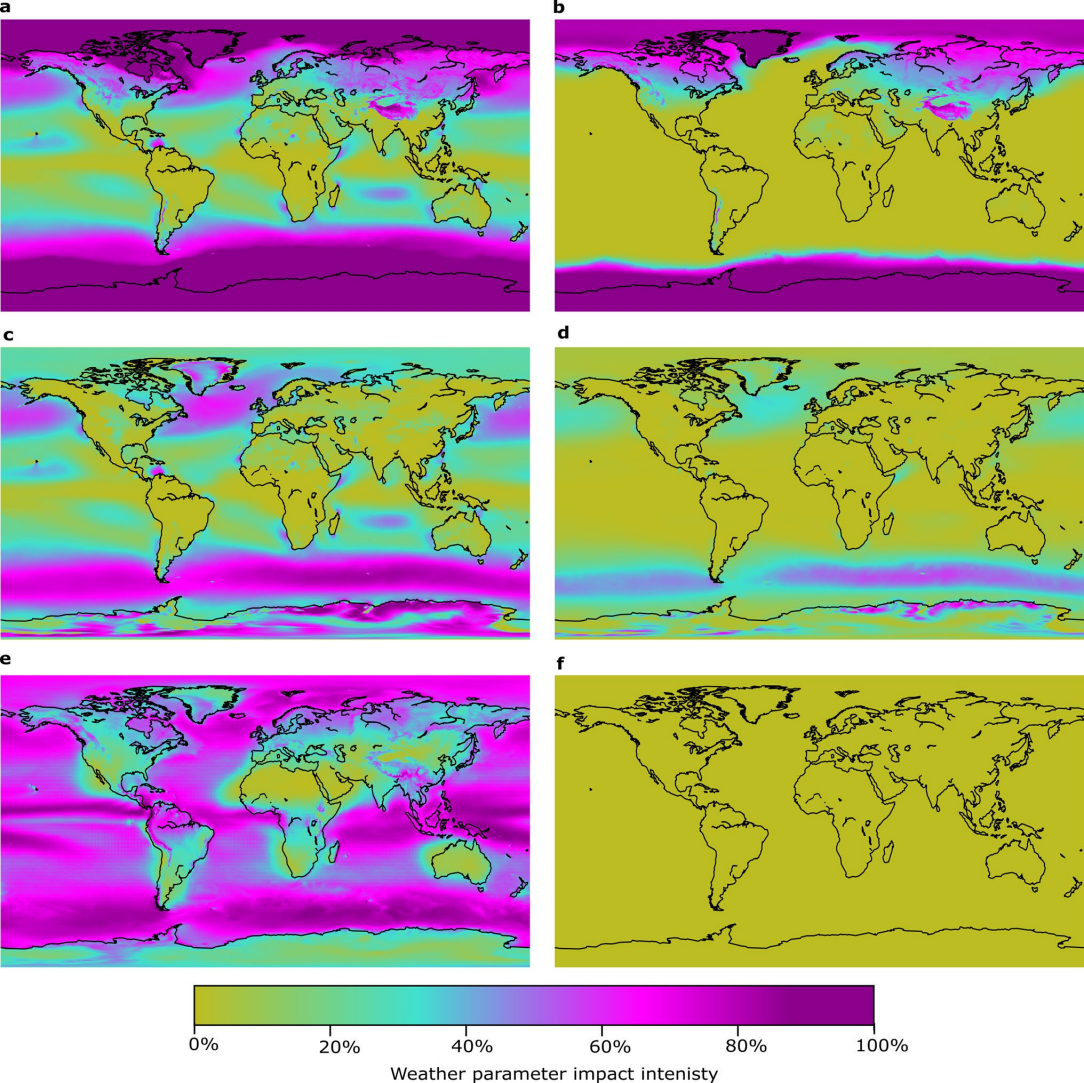
Global relative impact intensity of weather variables showing the impact of temperature on the flyability of CD (**a**) and WRD (**b**); the impact of wind speed on the flyability of CD (**c**) and WRD (**d**); and the impact of precipitation on the flyability of CD (**e**) and WRD (**f**). Impact intensity is the percentage of time that a weather variable prevents flight, independent of other weather variables.

The relative impact of each weather variable on drone flyability varies globally (Fig. 3). Impact intensity is the percentage of time that a weather variable prevents flight, independent of other weather variables. Precipitation has the highest median impact for the CD (30.2%) and the lowest median impact for the WRD (0%) over land, highlighting the potential to engineer drones to overcome this constraint. In general, the impact of precipitation is lower over land and lowest over desert regions, such as the Sahara and much of Australia. For the CD, precipitation impact can exceed 50% in tropical maritime countries. WRDs are most affected by temperature (median impact over land of 46.1%), especially near the poles. However, both types of drones experience minimal temperature constraints near the equator. For the WRD, land masses are generally unaffected, aside from high-latitude and high-elevation regions. Wind has the lowest relative impact on the CD (7.1% over land). Wind is more limiting on flyability over oceans. Over land, most of the world’s windiest regions have impact intensities < 20% (e.g., Central Great Plains of North America). Rare exceptions include Greenland and parts of southern Argentina, which are susceptible to high winds and demonstrate a high impact intensity. With a median impact intensity of 0.3% over land, WRDs are largely unaffected by wind.

### Temporal variability

**Figure 4:**
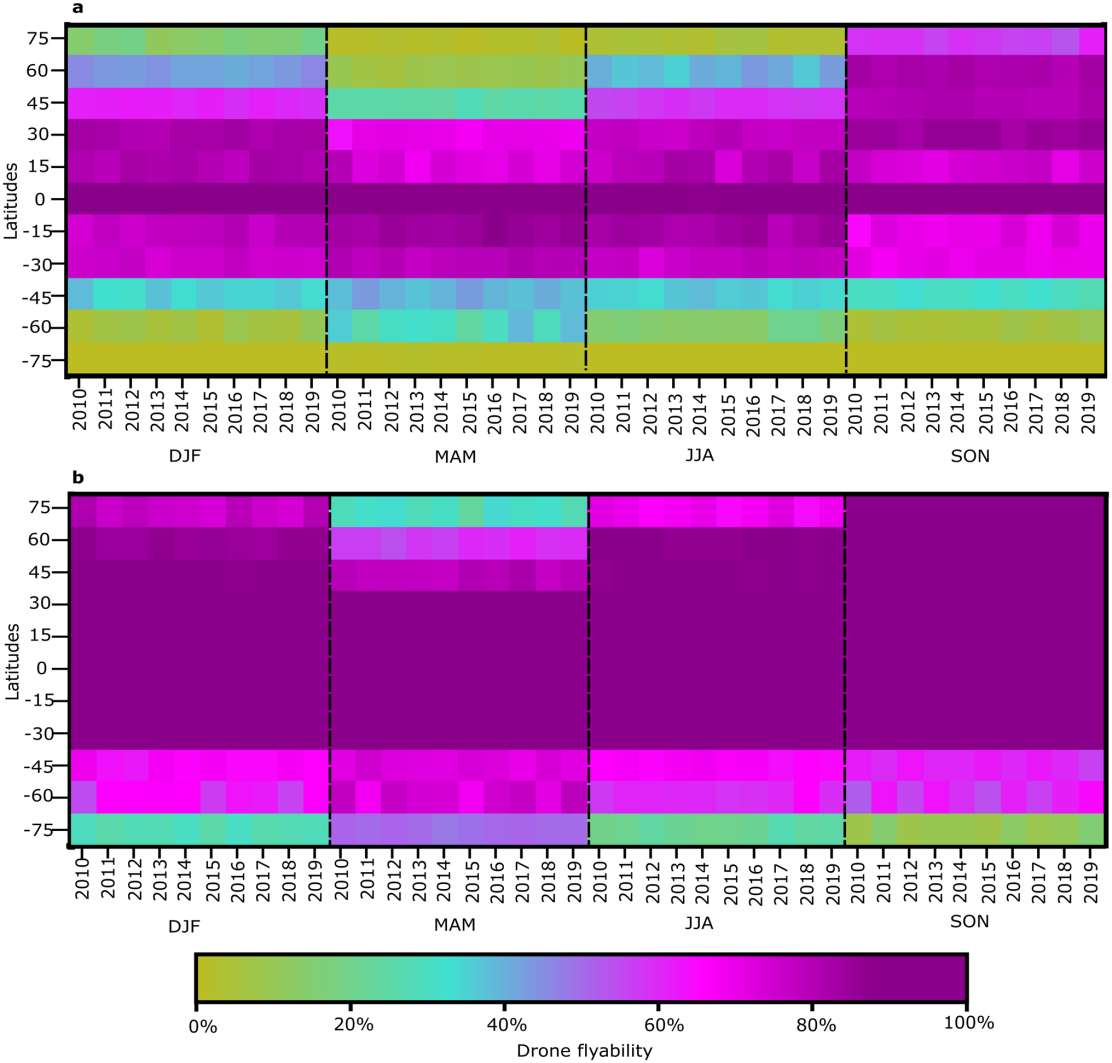
Flyability by year and season for CD (**a**) and WRD (**b**) at select latitudes (day-and-night). *DJF* December–January–February, *MAM* March–April–May, *JJA* June–July–August, *SON* September–October–November.

Flyability also varies temporally (Fig. 4). Interannual variability of flyability is minimal for a given season at a particular latitude (Fig. 4), with a mean year-to-year flyability change of 4.2% and maximum difference of 11% in December–January–February (DJF) 2015 and 2016 at 60° S. Seasonal variability has a greater impact on flyability than inter-annual variability, particularly at higher latitudes. For example, CD flyability at 60° N is > 80% in June–July–August (JJA), ~ 40% in March–April–May (MAM) and September–October–November (SON), and ~ 10% in DJF (Fig. 4a). Similar seasonal effects impact WRDs (Fig. 4B), but the influence is comparatively subdued with SON and MAM flyability > 80% and DJF flyability ~ 60% for the same latitude. Seasonal changes of flyability at high northern latitudes are greater than at high southern latitudes due to land/ocean contrasts, which are more pronounced in the northern hemisphere. Between 30° N and 30° S, where 51 of the top 100 most populous cities are located, flyability is highest near the equinoxes.

### Flyability benchmarks

**Figure 5:**
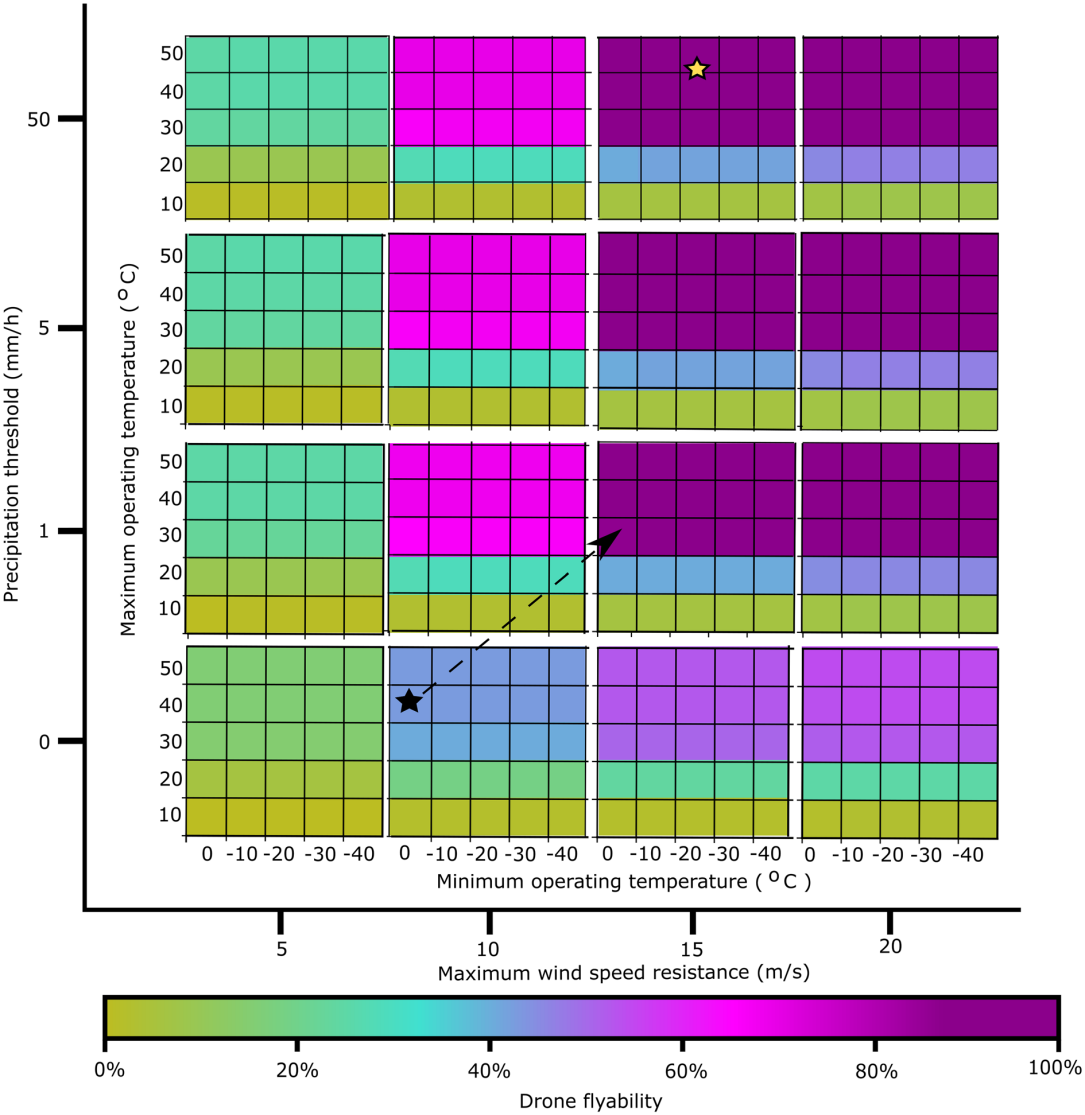
Multivariate matrix of drone flyability as influenced by air temperature, wind speed, and precipitation in the 100 most populated world cities^21^. Each interior plot shows median flyability as a function of minimum and maximum temperature tolerances. The vertical position of interior plots corresponds to precipitation thresholds, the horizontal position of interior plots corresponds to maximum wind speed resistance. The stars correspond to the CD (black) and WRD (yellow) to benchmark potential flyability gains. The arrow shows the greatest opportunity for improving flyability for CDs—increasing precipitation threshold by 1 mm/h and maximum wind speed resistance by 5 m/s.

To determine the level of weatherproofing necessary to increase median flyability, we invert the analysis and plot the intersection of the three weather variables from the 100 most populous cities, examining the thresholds for flyability gains (Fig. 5). The biggest gains can be achieved by developing drones with ingress protection to tolerate a precipitation rate of 1 mm/h and adding design and control measures to tolerate wind speeds up to 15 m/s at a flight altitude of 100 m. For the CD, these changes result in an increase of flyability from 41 to 87%. Weatherproofing beyond these levels, unless for specific use cases or more extreme climates, may be unnecessary. Increasing drone tolerance to extreme temperature results in minimal gains because the majority (*n* = 65) of the 100 most populous cities are in tropical or temperate climates. However, temperature has a much greater effect on flyability at high latitudes (Fig. 3), so increasing drone tolerance to lower temperatures can yield important flyability gains in these climates.

## Discussion

Weather has a strong impact on drone flyability, especially for CDs. Our results of limited CD flyability reflect similarities to findings recently presented by Lundby et al.^14^ and extended into a global context. Global drone uptime for on-demand applications is limited by weather and our analysis suggests that weather is an important global consideration for using drones in any on-demand applications. Parts of the world where on-demand drone operations need to meet service targets may require backup capacity. The most limiting weather is precipitation, but our analysis suggests the global geography of weather is highly diverse and not easy to generalize. In broad terms, the opportunities for on-demand drone use exist in the arid regions of the world where a combination of limited precipitation, low wind speeds, and benign temperatures can lead to high uptime and reliable operations.

Flyability is a metric of the time that drones can fly; however, flyability does not quantify the impact that these restrictions have on people and economics, which is not consistent globally. For example, flyability restrictions for delivery applications have higher per capita impact in locations with high population density. In contrast, flyability restrictions on the open ocean would have relatively small impact. One approach to proxy the impact of flyability restrictions for on-demand applications that serve people is to multiply non-flyable hours by population. To explore this, we calculate population impact for the 100 most populous cities (Supplementary Data 1). For example, the highest impacts in the CD class occur in megacities such as Kitakyushu-Fukuoka M.M.A., Haerbin, and Madras (15596.4, 13124.3, 10752.5 people h/year × 10^3^)—although these cities lack prolonged extreme weather. While population impact helps provide a generalization, application-specific modeling incorporating the myriad of variables that define ‘impact’ is important future work.

Results from the inverse analysis indicate that relatively small changes in CD tolerances to temperature, wind, and precipitation may have important effects on flyability (Fig. 5). For example, increases in the precipitation threshold and maximum wind speed limit of CD to 1 mm/h and 15 m/s, respectively, result in large flyability gains. In general, drones are not limited by high air temperatures. However, CD flyability can be increased by reducing the minimum operating temperature. The chemistry of lithium batteries commonly used in drones is affected by low temperatures^11,23^, so reducing minimum operating temperatures may require more advanced batteries or a better understanding of range limitations at low air temperatures^24^. As extreme low temperatures are generally limited to regions with very low population density, changes to drone power supply may be locally implemented if economically feasible. This noted, our study is generalized and global in scope. Specific applications will benefit from follow-up studies examining certain portions of the analysis space.

For drone applications and services in densely populated regions, our results show that the most important flyability gains can be achieved by adding liquid ingress protection or other measures to increase water resistance and by increasing drone power or improving aerodynamics to withstand higher wind speeds. Precipitation and condensation not only affect airworthiness, they can also affect other components and reduce the effectiveness of application-specific drone operations where optical sensors and cameras used for navigation, sense and avoid, and imaging are compromised by the accumulation of water or ice^10,14^. Ingress protection is effective in limiting water penetration based on international standards, but it does not guarantee that crashes will be prevented^25^ or protect against icing caused by freezing drizzle or supercooled droplets, which also affects airworthiness and causes drone crashes^26,27^. Icing conditions are not included in our analysis or mentioned in most drone manuals but will further reduce flyability for CDs and WRDs at higher latitudes where favourable icing conditions occur more frequently^28,29^. Piloted aircraft have numerous engineering controls and protocols to avoid deleterious effects of icing on flight characteristics, yet these and other weather conditions continue to result in flight delays and cancellations^30^. Thus, even with defensible ingress protection measures, some weather conditions will be unsuitable for safe drone operations.

IP ratings also indicate a level of protection against solid particles. This is important, as dust and coarser particles such as sand can clog the propeller motors and prevent flyability^25^. We note that the majority of CDs did not have ingress protection, and some manuals provided explicit warnings against allowing dust or sand to enter the motors. Most of the weather-resistant drones have IP ratings, although none had the “dust-tight” rating of 6X. The impact of small airborne particles such as dust and sand was not included in our analysis, though it is a considerable constraint on flyability, especially in arid and semi-arid regions such as northern Africa, the Arabian Peninsula, central Asia, northwestern China, Australia, and parts of North America.

Our global assessment of flyability depends on manufacturer-reported weather tolerances and weather reanalysis data. These data provide a continuous, pre-validated, and internally consistent gridded representation of the state of the atmosphere at relatively coarse spatial resolution (0.25 × 0.25°). Our analysis is limited to three common weather hazards that primarily denote adverse conditions^10^ and is not inclusive of all variables or current local conditions. Sub-grid variations of surface roughness, landcover, and topography can produce a wide variety of localized weather phenomena and variability not captured by reanalysis data, such as thermally- and density-driven wind, wind gusts, localized convective precipitation, icing conditions, cloud coverage, haze, fog, and dust/sand storms^10,14^. Site-specific studies should therefore be conducted to estimate and evaluate the safety risks presented by current local weather conditions for drone(s) intended to be used in an operation. Development of higher resolution weather forecasting technology to support drone operations is likely necessary in the near future to properly assess risk and safety of local drone operations^11,13^. In addition to weather, other flyability-constraining environmental parameters (which are commonly addressed in user manuals) that should be considered on a site-specific basis include: altitude above sea level, which relates to air density and will therefore affect aeronautical lift and, subsequently, drone endurance^10^; the presence of high levels of electromagnetism, large metal structures, and tall structures—all of which could interfere with onboard sensors^16^; and the stability of takeoff and landing locations (e.g., moving boats or trucks), which could impose additional challenges^16^.

Manufacturer-reported weather tolerances are another source of uncertainty^13^. We found no evidence of test protocols or certification standards in manufacturer-provided drone manuals or in drone regulations. We also dismissed drones without documented guidelines for any of the three weather variables, rather than estimating tolerances as done in previous studies^14^. Furthermore, the precise meaning of wind tolerance and whether the value refers to sustained wind speed or gusts is an open question. Without a defensible set of tests and criteria for performance evaluation, it is difficult to legally or practically guarantee that any drone will meet currently stated weather tolerances. While our results inform strategic efforts to increase drone flyability in highly populated regions, we note that a current lack of industry and regulatory standardization of testing and verification of weather tolerances may hinder such developments.

The aeronautical industry—through a ‘culture of safety’—has created a mature and rigorous system for airworthiness performance evaluation that is widely understood to be effective. In comparison, most small drones currently used in commercial applications and services are built from commodity parts that are not subject to the same aeronautical-grade standards or performance testing. This has been beneficial to the drone industry, which has effectively implemented many advances at a rate that would be difficult with the rigorous checks and balances in aerospace. However, research has shown that most safety occurrences with drones involve system component failures^31^. To mature drone operations for commercial on-demand applications, particularly in close proximity to people or property, a defensible and standard set of weather performance tests for drones will be necessary.

## Methods

### Overview

Drone flyability was assessed by calculating the mean proportion of the year with suitable weather conditions for drone flight. We used manufacturer-provided weather constraints for popular commercially registered drone models and hourly historical meteorological reanalysis data spanning ten years. In this analysis, a flyable timestep was defined as one hourly instance in which a given drone had specifications allowing flight, measured with three weather factors: air temperature, wind speed, and precipitation. Drone flyability was analyzed globally using a geographical grid: for each grid cell and timestep, the mean air temperature (°C), wind speed (m/s), and precipitation (mm/h) were compared to the operational temperature range (°C), maximum wind speed resistance (m/s), and precipitation tolerance (mm/h) of the drone. In addition to weather-constrained flyability, we also considered daylight constraints, where flyability was contingent on suitable weather conditions and the presence of daylight. Finally, we performed an inverse analysis that quantified flyability for different combinations of weather tolerance parameter values.

### Drone weather constraints

Weather-based operational constraints for small drones (i.e., those with a maximum takeoff weight < 25 kg) were obtained for the most popular commercially registered drones in the US, which were identified using registration data from the FAA. According to the FAA, as of 05 May 2020, there were 365,053 small drones actively registered for commercial operations (i.e., under 14 Code of Federal Regulations [CFR] Part 107^32^). We obtained a list of the 500 most popular drone models in terms of the number of active registrations, which represented 85% (*n* = 310,611) of the total active registrations.

From this list, we identified the 50 most frequently registered drone models, which represented 69% (*n* = 250,747) of the total active registrations. We searched for weather tolerance data using manufacturer-issued information sources (i.e., manuals, brochures, websites, and direct correspondence via email). The weather tolerance data fields included minimum and maximum operating temperature (°C), maximum wind speed resistance (m/s), and precipitation tolerance (mm/h). For operating temperature and maximum wind speed resistance, only quantitative descriptions were usable in the analysis. For example, qualitative descriptions such as “this aircraft cannot be operated in strong winds” were not usable. For precipitation tolerance, qualitative descriptions were only usable for indicating a 0 mm/h precipitation tolerance (e.g., “this aircraft cannot be operated in rain”), while quantitative descriptions were necessary for specifying precipitation tolerances greater than zero.

After aggregating rows pertaining to the same drone model (but containing various model names), the top 50 most frequently registered drones were reduced to 33 unique entries. Of the 33 unique drone models on the list, 73% (*n* = 24) contained manufacturer-provided weather tolerance data for all four fields (and thus were included in the flyability analysis), while 18% (*n* = 6) did not contain data for all four fields, and 9% (*n* = 3) contained model names for which no information was found. Using the 24 drone models containing data for all four weather tolerance fields, we extracted the median from each field: 0 °C for minimum operating temperature; 40 °C for maximum operating temperature; 10 m/s for maximum wind speed resistance; and 0 mm/h for precipitation tolerance. We termed this group of median values as the CD class, which represents 50% (*n* = 12) of the 24 drone models used for the median calculations. Table 1 lists the drone models for which all four weather tolerance parameters were found, and Supplementary Data 2 contains the full list of drone models, their quantitative and qualitative manufacturer-provided weather tolerance data, and data sources.

**Table 1:** Condensed list of top small-drone commercial registrations in the United States as of 05 May 2020.

Total FAA registrations (14 CFR Part 107)	Manufacturer	Model	Min. operating temp. (°C)	Max. operating temp. (°C)	Max. wind speed resistance (m/s)	Precipitation tolerance (mm/h)^a^
47,964	DJI	Mavic Pro	0	40	10	0.00
8493	DJI	Mavic Pro Platinum	0	40	10	0.00
37,462	DJI	Mavic Air	0	40	10	0.00
2007	DJI	Mavic 2	− 10	40	10	0.00
29,789	DJI	Mavic 2 Pro	− 10	40	10	0.00
7945	DJI	Mavic 2 Zoom	− 10	40	10	0.00
847	DJI	Mavic 2 Enterprise	− 10	40	10	0.00
1360	DJI	Mavic 2 Enterprise Dual	− 10	40	10	0.00
1816	DJI	Mavic Mini	0	40	8	0.00
7629	DJI	Phantom 3 Standard	0	40	10	0.00
1155	DJI	Phantom 3 Advanced	0	40	10	0.00
3647	DJI	Phantom 3 Professional	0	40	10	0.00
9023	DJI	Phantom 4	0	40	10	0.00
1836	DJI	Phantom 4 Advanced	0	40	10	0.00
17,323	DJI	Phantom 4 Pro	0	40	10	0.00
929	DJI	Phantom 4 Pro+	0	40	10	0.00
3422	DJI	Phantom 4 Pro V2.0	0	40	10	0.00
30,757	DJI	Spark	0	40	10	0.00
2267	DJI	Inspire 1	− 10	40	10	0.00
4470	DJI	Inspire 2	− 20	40	10	0.00
980	DJI	Matrice 210	− 20	45	12	10.00
3043	Parrot	Bebop 2	5	40	17	0.00
1987	Parrot	Anafi	− 10	40	14	0.00
1815	3DR	Solo	0	45	11	0.00

^a^Precipitation tolerance values of 0.00 mm/h were derived from qualitative descriptions (e.g., “this aircraft cannot be operated in rain”).

For comparison, we also defined a weather-resistant drone (WRD) class, which represents drones that are operable in rain, have wider operating temperature ranges, and can resist higher wind speeds. Because all but one drone model in Table 1 had a precipitation tolerance of 0.00 mm/h, we searched through the full list of the 500 most frequently registered drones and identified those that are operable in rain using manufacturer-provided information sources. Table 2 shows weather tolerance data for 8 drones that are operable in rain and contained manufacturer-provided weather tolerance data for all four fields. From these drones, we chose the Zipline Zip to represent the WRD class because it is the only drone advertised and used for delivery purposes. Thus, the values for each weather tolerance data field were: − 20 °C for minimum operating temperature; 46 °C for maximum operating temperature; 14 m/s for maximum wind speed resistance; and 50 mm/h for precipitation tolerance.

**Table 2:** Small drones commercially registered in the United States as of 05 May 2020 with precipitation tolerance > 0.00 mm/h.

Total FAA registrations (14 CFR Part 107)	Manufacturer	Model	Min. operating temp. (°C)	Max. operating temp. (°C)	Max. wind speed resistance (m/s)	Precipitation tolerance (mm/h)
647	DJI	Matrice 200	− 20	45	12	10.00
429	DJI	Matrice 210	− 20	45	12	10.00
161	DJI	Matrice 210 RTK	− 20	45	12	10.00
273	DJI	Matrice 200 V2	− 20	50	12	10.00
378	DJI	Matrice 210 V2	− 20	50	12	10.00
102	DJI	Matrice 210 RTK V2	− 20	50	12	10.00
48	Lockheed Martin	Indago 3.1	− 34.44	48.89	11.18	6.99
150	Zipline	Zip	− 20	46	14	50.00

### Meteorological data

Meteorological data were derived from the ERA5 reanalysis dataset developed by the European Centre for Medium-Range Weather Forecasts^19,20^. This dataset models global meteorological parameters from historical data records spanning 1979 to present day. Meteorological attributes are available at a spatial resolution of 0.25 × 0.25°, approximately 27.25 km (at the equator), and an hourly temporal resolution (24 timesteps per day), resulting in a global dataset comprised of 1,038,240 grid cells per timestep. For this analysis, we obtained complete daily datasets from 01 January 2008 to 31 December 2017 and determined drone flyability based on 2 m air temperature (*2 m temperature*), 100 m wind speed (*100 m u-component of wind* and *100 m v-component of wind*), and total precipitation (*total precipitation*) variables. The *2 m temperature* parameter is an instantaneous modeled estimate of the air temperature (in Kelvin, K) at 2 m above Earth’s surface (i.e., land, sea, or inland waters), which is spatially averaged within each grid cell. The *100 m u-component of wind* and *100 m v-component of wind* parameters are instantaneous modeled estimates of the eastward and northward wind speed (m/s), respectively, 100 m above Earth’s surface and spatially averaged within each grid cell. The *total precipitation* parameter represents the depth (m) of liquid water (derived from modeled rain and snow estimates) accumulating over each hourly timestep and spread evenly within each grid cell^20^.

Adjustments were made to raw data values from the ERA5 dataset to ensure compatibility for comparison with drone specifications. Air temperature was converted from K to °C by subtracting 273.15. Since raw values of wind data in the ERA5 dataset are recorded as two separate bilinear speed vectors, we calculated the mean wind speed for each grid cell and timestep combination from these vectors using Pythagorean theorem:

1 $${MWS}_{i,j}= \sqrt{{{u}_{i,j}}^{2}+{{v}_{i,j}}^{2}}$$

where ${MWS}_{i,j}$ is the mean wind speed (m/s) at grid cell $i$ and timestep $j$, $u$ is the eastward wind speed component (m/s), and $v$ is the northward wind speed component (m/s). Total precipitation was converted from units of m to mm and rounded to two decimal places. Thus, timesteps with total precipitation values < 0.005 mm/h were considered precipitation-free.

### Daylight constraints on drone flyability

Daylight is an important factor that determines drone flyability for certain drone models, applications, and certifications. Therefore, an additional analysis was performed using the weather factors plus an additional parameter specifying whether daylight was present or absent within each grid cell and timestep combination. Daylight presence was determined based on the solar elevation angle:

2 $${SEA}_{i,j} = \frac{{SA}_{i,j} \times 180}{\pi }$$

where ${SEA}_{i,j}$ is the solar elevation angle (degrees) at grid cell $i$ and timestep $j$, and ${SA}_{i,j}$ represents the solar altitude (degrees) at grid cell $i$ and timestep $j$. The solar elevation angle indicates the angle between the horizon and the center of the solar disc. It also relates to the twilight phase on Earth. There are three different twilight phases that are usually applied to define the day and night boundary (i.e., the presence or absence of daylight): civil twilight, nautical twilight, and astronomical twilight. We used civil twilight because FAA Federal Aviation Regulations define night as “the time between the end of evening civil twilight and the beginning of morning civil twilight”^32^. Morning and evening civil twilight correspond to a solar elevation angle of 6° below the horizon (− 6°)^32^. Based on this definition, we created a rule for distinguishing between daytime and nighttime: daylight was present if ${SEA}_{i,j}$ was greater than − 6°.

To obtain ${SA}_{i,j}$ for the calculation of ${SEA}_{i,j}$, each timestep within each grid cell was converted from Coordinated Universal Time (UTC) to local time. First, the longitude of each grid cell was used to determine its standard time zone:

3 $$STZ_{i} = \left\{ {\begin{array}{*{20}l} {quotient\left( {\frac{{longitude_{i} }}{15}} \right) + 1,} \hfill & {if\;remainder\left( {\frac{{longitude_{i} }}{15}} \right) > 7.5} \hfill \\ {quotient\left( {\frac{{longitude_{i} }}{15}} \right),} \hfill & {if\;remainder\left( {\frac{{longitude_{i} }}{15}} \right) \le 7.5} \hfill \\ \end{array} } \right.$$

where ${STZ}_{j}$ is the standard time zone of grid cell $i$, and $l{ongitude}_{i}$ is the longitude of the center of grid cell $i$. Then, we calculated the local time for each grid cell and timestep combination:

4 $$LT_{i,j} = \left\{ {\begin{array}{*{20}l} {12 - \left( {0 - UTC_{i,j} } \right) + 24,} \hfill & {if\;12 - \left( {0 - STZ_{i} } \right) < 0} \hfill \\ {12 - \left( {0 - UTC_{i,j} } \right) - 24,} \hfill & {if\;12 - \left( {0 - STZ_{i} } \right) > 24} \hfill \\ {12 - \left( {0 - UTC_{i,j} } \right),} \hfill & {if\;0 \le 12 - \left( {0 - STZ_{i} } \right) \le 24} \hfill \\ \end{array} } \right.$$

where ${LT}_{i,j}$ is the local time of grid cell $i$ and timestep $j$, and ${UTC}_{i,j}$ is the time in UTC of grid cell $i$ and timestep $j$. The local time was then converted to solar hour angle, which is the angular displacement of the sun east or west of the local meridian due to Earth’s rotation (15°/h)^33^:

5 $${SHA}_{i,j} =({LT}_{i,j}-12)\times 15$$

where ${SHA}_{i,j}$ is the solar hour angle (°) of grid cell $i$ and timestep $j$. The solar hour angle was then converted to solar altitude angle, which is the angle between the horizon plane and the solar beam^32^ ﻿at the grid cell center:

6 $$\text{sin}({SAA}_{i,j})=\text{sin}({latitude}_{i})\text{sin}({SDA}_{j})+\text{cos}({latitude}_{i})\text{cos}({SDA}_{j}){\text{cos}}({SHA}_{i,j})$$

where ${SAA}_{i,j}$ is the solar altitude angle (°) of grid cell $i$ and timestep $j$, ${latitude}_{i}$ is the latitude of the center of grid cell $i$, and ${SDA}_{j}$ is the solar declination angle of timestep $j$. The solar declination angle^32^ was calculated for each timestep:

7 $${SDA}_{j}=23.45sin\left[\frac{360}{365}(284+{N}_{j})\right]$$

where ${N}_{j}$ is the day of the year corresponding to timestep $j$. Finally, the solar altitude angle was converted to the solar elevation angle (${SEA}_{i,j}$) to determine if daylight was present for grid cell $i$ and timestep $j$.

### Global flyability and spatial and temporal variability

**Figure 6:**
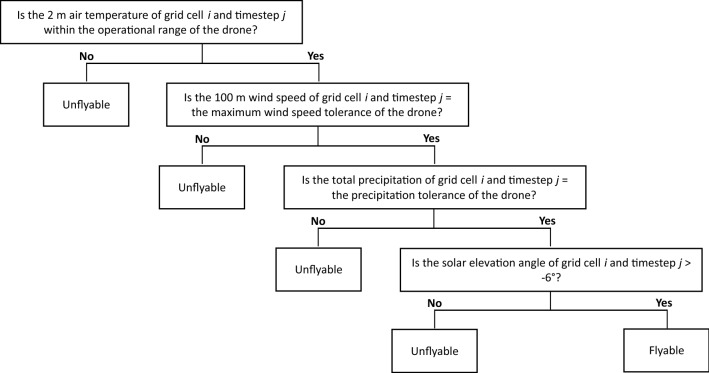
Binary decision diagram used to determine if the weather conditions corresponding to each grid cell and timestep combination were suitable for drone flight for the common and weather-resistant drone classes. If all meteorological requirements were met, the grid cell and timestep combination was counted as one flyable timestep. The last decision rule based on daylight was included in the additional analysis.

Drone flyability was expressed as the mean proportion of the year (i.e., mean annual percentage of timesteps) with suitable conditions for drone flight. To count flyable timesteps, we constructed a binary decision diagram (Fig. 6) to sequentially determine if a given grid cell and timestep combination satisfied all weather tolerance values for each drone class. For each grid cell, flyable timesteps were summed, divided by the number of years of analysis (*n* = 10), and converted to a percentage of the total number of timesteps per year. Global flyability was quantified as the median value of all grid cells, which was compared among the CD and WRD classes, with and without daylight constraints. Flyability was also examined among the 100 most populated world cities (according to projected population values for 2020 from the United Nations^21^). For each city, the ERA5 grid cell containing the city center location was used to calculate flyability. Temporal and spatial variations in flyability were assessed among four seasons (01 Sep to 30 Nov; 01 Dec to 28/29 Feb; 01 Mar to 31 May, and 01 Jun to 31 Aug) at eleven latitudes (0°, 15° N, 15° S, 30° N, 30° S, 45° N, 45° S, 60° N, 60° S, 75° N, and 75° S).

### Weather parameter impact intensity

Each weather parameter was examined in isolation to calculate its negative impact on flyability. For each parameter, we calculated the percentage of timesteps in each grid cell that were categorized as unflyable due to the ERA5 parameter value exceeding the corresponding drone tolerance value or range. This assessment was performed individually for each parameter (not sequentially as shown in Fig. 6), so a given grid cell and timestep combination could be categorized as unflyable by multiple weather parameters.

### Inverse analysis

To quantify flyability for different combinations of drone weather tolerance parameter values, we performed an inverse analysis. We used the same ERA5 data range (01 January 2008 to 31 December 2017) and parameters (*2 m temperature*, *100 m u-component of wind*, *100 m v-component of wind*, *total precipitation)* as in the primary analyses. We considered 400 combinations of tolerance parameter values and quantified the median annual flyability for each combination based on the top 100 most populated world cities^21^. The tolerance parameter values were: − 40, − 30, − 20, − 10, and 0 °C (minimum temperature); 10, 20, 30, 40, and 50 °C (maximum temperature); 5, 10, 15, and 20 m/s (maximum wind speed); and 0, 1, 5, and 50 mm/h (precipitation threshold).

### Methodological limitations

The limitations of this analysis correspond to the drone weather tolerances, weather reanalysis data, and their comparison to quantify flyability. The list of drone models we used to represent CDs and WRDs was not exhaustive; we considered the top 50 (for CDs) and top 500 (for WRDs) drone models based on the number of active commercial registrations of small UAS (< 25 kg) in the United States, which represented 69% (for CDs) and 85% (for WRDs) of total active commercial registrations as of 05 May 2020. There are likely other drone models that fit within the CD and WRD classes, but they were excluded from the analysis due one or more of the following: (1) at least one weather constraint parameter was not specified by the manufacturer; (2) the drone’s FAA commercial registration rank was higher than 50 (for CDs) and 500 (for WRDs); and (3) the drone was not commercially registered in the United States. Our analysis did not consider drone models that are unregistered or registered for recreational use in the United States, nor did we include drone models that are unregistered or registered in other countries.

The weather constraints representing the CD and WRD classes were directly compared to ERA5 weather data to quantify flyability, though there are several limitations associated with this comparison. The ERA5 parameters are generated by combining instantaneous modeled estimates that are spatially averaged within each grid cell and direct observations at point locations that reveal localized variations. Within a grid cell, there could be local weather conditions exceeding the operating envelope of a CD or WRD. However, due to the modeling and spatial resolution of ERA5 data, such conditions preventing flyability may not be represented. Furthermore, the weather parameters are inconsistent in terms of the altitudes at which they are modeled: 2 m, 100 m, and surface level for the air temperature, wind speed, and precipitation parameters, respectively. The 100 m wind speed data were used in the analysis (instead of ERA5 10 m wind speed data) because this altitude is more representative of drone operations based on factors such as areal coverage requirements for surveying, collision avoidance with vertical objects, and nationally mandated maximum flight altitudes for drones. Ideally, all three weather parameters would be modeled at 100 m. In addition to the three weather parameters and daylight parameter considered in this analysis, there are other parameters that constrain drone flyability, such as icing, fog, visibility, smoke, and dust. If additional constraint factors are considered, drone flyability estimates would likely be reduced. In summary, the flyability analysis is limited by: (1) the modeling and spatial resolution of the ERA5 data; (2) the inconsistent altitudes at which the ERA data parameters are modeled; and (3) the exclusion of additional factors that constrain drone flyability.

## Supplementary Information

Supplementary Legends.

Supplementary Information 1.

Supplementary Information 2.

## Data Availability

The datasets analyzed during the study, if not in the “Supplementary Data”, are available from the corresponding author on reasonable request.

## Code availability

The weather data processing and analysis were programmed in Python with standard packages. The results can be reproduced by employing the equations, explanation, and parameters provided in the main text. Additional code and data will be made available upon request.

## References

[1] Giones F, Brem A (2017). From toys to tools: The co-evolution of technological and entrepreneurial developments in the drone industry. Bus. Horiz..

[2] Floreano D, Wood RJ (2015). Science, technology and the future of small autonomous drones. Nature.

[3] Magistretti S, Dell’Era C (2019). Unveiling opportunities afforded by emerging technologies: Evidence from the drone industry. Technol. Anal. Strat. Manag..

[4] Teal Group. World Civil Unmanned Aerial Systems Market Profile & Forecast (2017). http://tealgroup.com/images/TGCTOC/WCUAS2017TOC_EO.pdf. (Accessed 23 October 2020).

[5] US Federal Aviation Administration (FAA). UAS by the Numbers (2020). https://www.faa.gov/uas/resources/by_the_numbers/. (Accessed 23 October 2020).

[6] Merkert R, Bushell J (2020). Managing the drone revolution: A systematic literature review into the current use of airborne drones and future strategic directions for their effective control. J. Air Transp. Manag..

[7] Prevot, T., Rios, J., Kopardekar, P., Robinson, J. E. III, Johnson, M., Jung, J. UAS Traffic Management (UTM) concept of operations to safely enable low altitude flight operations. in* 16th AIAA Aviation Technology, Integration, and Operations Conference*. 2016–3292. Reston, VA: AIAA (2016). https://utm.arc.nasa.gov/docs/Kopardekar_2016-3292_ATIO.pdf. (Accessed 23 October 2020).

[8] Hatfield M, Cahill C, Webley P, Garron J, Beltran R (2020). Integration of unmanned aircraft systems into the national airspace system-efforts by the university of alaska to support the FAA/NASA UAS traffic management program. Remote Sensing.

[9] Watkins S, Burry J, Mohamed A, Marino M, Prudden S, Fisher A, Kloet N, Joakobi T, Clothier R (2020). Ten questions concerning the use of drones in urban environments. Build. Environ..

[10] Ranquist, E., M. Steiner, & Argrow, B. Exploring the range of weather impacts on UAS operations. in *18th Conference on Aviation, Range, and Aerospace Meteorology*, Seattle, WA, American Meteorological Society, J3.1. (2017). https://ams.confex.com/ams/97Annual/webprogram/Paper309274.html. (Accessed 23 October 2020).

[11] Roseman CA, Argrow BM (2020). Weather hazard risk quantification for sUAS safety risk management. J. Atmos. Oceanic Tech..

[12] American National Standards Institute (ANSI). Standardization Roadmap For Unmanned Aircraft Systems, Version 1.0. ANSI Unmanned Aircraft Systems Standardization Collaborative (2018). https://share.ansi.org/Shared%20Documents/Standards%20Activities/UASSC/ANSI_UASSC_Roadmap_December_2018.pdf. (Accessed 23 October 2020).

[13] Roseman, C. A., Argrow, B. M., Pinto, J. O. Targeted weather forecasts for small unmanned aircraft systems. in *19th Conf. on Aviation, Range, and Aerospace Meteorology, Phoenix, AZ, Amer. Meteor. Soc*., 1.4. (2019). https://ams.confex.com/ams/2019Annual/webprogram/Paper351492.html. (Accessed 23 October 2020).

[14] Lundby, T., Christiansen, M.P., Jensen, K. Towards a weather analysis software framework to improve UAS operational safety. in *2019 International Conference on Unmanned Aircraft Systems (ICUAS)*, 1372–1380. IEEE (2019).

[15] Glasheen, K., Pinto, J., Steiner, M., Frew, E.W. Experimental assessment of local weather forecasts for small unmanned aircraft flight. in *Aiaa scitech 2019 forum*, 1193. (2019).

[16] DJI. DJI Mavic Air 2 User Manual (2020). https://dl.djicdn.com/downloads/Mavic_Air_2/20200615/Mavic_Air_2_User_Manual_v1.2_en_.pdf. (Accessed 23 October 2020).

[17] Government of Canada. Canadian Aviation Regulations (CARs) and Standards. SOR/96-433 (2019). https://tc.canada.ca/en/corporate-services/acts-regulations/list-regulations/canadian-aviation-regulations-sor-96-433. (Accessed 23 October 2020).

[18] Newsweek. Drone Delivery for Amazon and Google Slowed by Headwinds (2014). https://www.newsweek.com/will-wind-be-end-commercial-drone-delivery-amazon-and-google-275999. (Accessed 23 October 2020).

[19] Hersbach H, Bell B, Berrisford P, Hirahara S, Horányi A, Muñoz-Sabater J, Nicolas J, Peubey C, Radu R, Schepers D, Simmons A, Soci C, Abdalla S, Abellan X, Balsamo G, Bechtold P, Biavati G, Bidlot J, Bonavita M, De Chiara G, Dahlgren P, Dee D, Diamantakis M, Dragani R, Flemming J, Forber R, Fuentes M, Geer A, Haimberger L, Healy S, Hogan RJ, Hólm E, Janisková M, Keeley S, Laloyaux P, Lopez P, Lupu C, Radnoti G, de Rosnay P, Rozum I, Vamborg F, Villaume S, Thépaut J-N (2020). The ERA5 global reanalysis. Q. J. R. Meteorol. Soc..

[20] ECMWF. ERA5 hourly data on single levels from 1979 to present. (2020). https://cds.climate.copernicus.eu/cdsapp#!/dataset/reanalysis-era5-single-levels?tab=overview. (Accessed 23 October 2020).

[21] Singh KK, Frazier AE (2018). A meta-analysis and review of unmanned aircraft system (UAS) imagery for terrestrial applications. Int. J. Remote Sens..

[22] United Nations, Department of Economic and Social Affairs, Population Division. World Urbanization Prospects: The 2018 Revision [File 12: Population of Urban Agglomerations with 300,000 Inhabitants or More in 2018, by Country, 1950–2035 (thousands)], Online Edition. (2018). https://population.un.org/wup/Download/. (Accessed 23 October 2020).

[23] Ji Y, Zhang Y, Wang C-Y (2013). Li-Ion cell operation at low temperatures. J. Electrochem. Soc..

[24] Kim SJ, Lim GJ, Cho J (2018). Drone flight scheduling under uncertainty on battery duration and air temperature. Comput. Ind. Eng..

[25] Air Accidents Investigation Branch (AAIB). AAIB Bulletin 1/2020 (2020). https://assets.publishing.service.gov.uk/government/uploads/system/uploads/attachment_data/file/919996/AAIB_Bulletin_1-2020_Hi_Res.pdf. (Accessed 23 October 2020).

[26] Cassano JJ, Seefeldt MW, Palo S, Knuth SL, Bradley AC, Herrman PD, Kernebone PA, Logan NJ (2016). Observations of the atmosphere and surface state over Terra Nova Bay, Antarctica, using unmanned aerial systems. Earth Syst. Sci. Data.

[27] Lampert A, Alstädter B, Bärfuss K, Bretschneider L, Sandgaard J, Michaelis J, Lobitz L, Asmussen M, Damm E, Käthner R, Krüger T, Lüpker C, Nowak S, Peuker A, Rausch T, Reiser F, Scholtz A, Sotomayor Zakharov D, Gaus D, Bansmer S, Wehner B, Pätzold F (2020). Unmanned aerial systems for investigating the polar atmospheric boundary layer—Technical challenges and examples of applications. Atmosphere.

[28] Bernstein BC, Wolff CA, McDonough F (2007). An inferred climatology of icing conditions aloft, including supercooled large drops. Part I: Canada and the continental United States. J. Appl. Meteorol. Climatol..

[29] Bernstein BC, Le Bot C (2009). An inferred climatology of icing conditions aloft, including supercooled large drops. Part II: Europe, Asia, and the globe. J. Appl. Meteorol. Climatol..

[30] Goodman CJ, Small Griswold JD (2019). Meteorological impacts on commercial aviation delays and cancellations in the continental United States. J. Appl. Meteorol. Climatol..

[31] Wild G, Gavin K, Murray J, Silva J, Baxter G (2017). A post-accident analysis of civil remotely-piloted aircraft system accidents and incidents. J. Aerosp. Technol. Manag..

[32] US Federal Aviation Administration (FAA). Electronic Code of Federal Regulations, 14 CFR 1.1. https://www.ecfr.gov/cgi-bin/retrieveECFR?n=14y1.0.1.1.1. (Accessed 23 October 2020).

[33] Kalogirou SA (2009). Solar Energy Engineering: Processes and Systems.

[34] NOAA. Solar Calculator Glossary. Global Monitoring Laboratory: Earth System Research Laboratories. https://www.esrl.noaa.gov/gmd/grad/solcalc/glossary.html. (Accessed 23 October 2020).

