# Author Correction: Weather constraints on global drone flyability

**DOI:** 10.1038/s41598-021-00537-7

**Published:** 2021-10-26

**Authors:** Mozhou Gao, Chris H. Hugenholtz, Thomas A. Fox, Maja Kucharczyk, Thomas E. Barchyn, Paul R. Nesbit

**Affiliations:** 1 Department of Geography, University of Calgary, 2500 University Drive NW, Calgary, AB T2N 1N4 Canada; 2 Department of Geoscience, University of Calgary, 2500 University Drive NW, Calgary, AB T2N 1N4 Canada

Correction to: *Scientific Reports* 10.1038/s41598-021-91325-w, published online 08 June 2021

The original version of this Article contained errors in Supplementary Data 1, where all the flyability values for global cities were extracted incorrectly. The original Supplementary Information 1 file is provided below.

As the result of the errors, statistics quoted in the Results section under the subheading ‘Global flyability’,

“We examine weather and daylight constraints for the 100 most populated world cities^22^ (Fig. 1). Median day-and-night flyability over these urban areas increases to 39.5% and 95.3% for CDs and WRDs, respectively, and drops to 13.5% and 51.7% in daylight-only operations (Supplementary Data 1). Flyability is extremely variable, ranging from 95.7% for CDs in Johannesburg, South Africa, to 2.3% in Saint Petersburg, Russia. Daylight-only flyability values for CDs and WRDs, respectively, range from 0.4 to 41.1% and 26.8 to 54.6%, while day-and-night flyability ranges from 2.3 to 95.7% and 48.5 to 100.0% (Supplementary Data 1). These large ranges demonstrate that weather-constrained flyability varies with geography and that flyability tends to be higher in densely populated regions than on a global basis.”

now reads:

“We examine weather and daylight constraints for the 100 most populated world cities^22^ (Fig. 1). Median day-and-night flyability over these urban areas increases to 64.3% and 99.5% for CDs and WRDs, respectively, and drops to 23.6% and 54.2% in daylight-only operations (Supplementary Data 1). Flyability is extremely variable for CDs, ranging from 94.8% in Alexandria, Egypt, to 29.8% in Singapore. Daylight-only flyability and day-and-night flyability ranges, respectively, from 4.5 to 39.6% and from 29.8 to 94.8% (Supplementary Data 1). These large ranges for CDs demonstrate that weather-constrained flyability varies with geography and that flyability tends to be higher in densely populated regions than on a global basis. Flyability is consistent for WRDs. Daylight-only flyability ranges from 49.5 to 59.0%, and day-and-night flyability ranges from 91.5 to 100.0% (Supplementary Data 1).”

Furthermore, the errors resulted in the incorrect Figure 1. The original Figure 1 and accompanying legend appear below.

**Figure 1:**
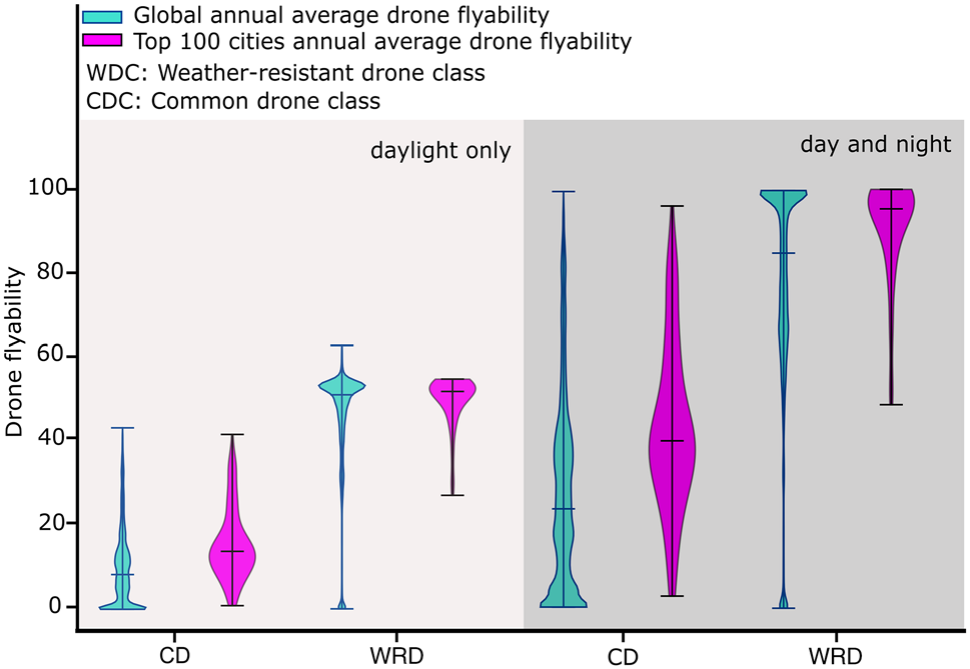
Violin plots of day-and-night and daylight-only flyability for CD and WRD. Cyan violins correspond to the distribution of flyabilities of CD and WRD globally and orange violins represent the distribution of flyabilities of CD and WRD from 100 most populated cities across the world^22^. The upper and lower hinges of violin plots indicate the minimum and maximum flyabilities. The horizontal bar of each violin represents the median of annual average drone flyability.

Finally, in the Discussion section,

“For example, the highest impacts in the CD class occur in megacities such as Delhi, Bombay, and Beijing (17,308.0, 15,093.5, 14,506.3 people h/year × 10^3^)— although these cities lack prolonged extreme weather.”

now reads:

“For example, the highest impacts in the CD class occur in megacities such as Kitakyushu-Fukuoka M.M.A., Haerbin, and Madras (15,596.4, 13,124.3, 10,752.5 people h/year × 10^3^)—although these cities lack prolonged extreme weather.”

The original Article and accompanying Supplementary Information file have been corrected.

## Supplementary Information

Supplementary Information.

