# Supplementary material for: Weather constraints on global drone flyability
Source: Sci Rep. 2021 Jun 8;11:12092. doi: 10.1038/s41598-021-91325-w (PMC8187708; doi:10.1038/s41598-021-91325-w)
Supplement: Supplementary file 1 — Supplementary Legends. [file 41598_2021_91325_MOESM1_ESM.docx]

**Supplementary Information for**

**Weather constraints on global drone flyability**

**Mozhou Gao^1^, Chris H. Hugenholtz^1,^*, Thomas A. Fox^1^, Maja Kucharczyk^1^, Thomas E. Barchyn^1^, Paul R. Nesbit^1^**

University of Calgary, Department of Geography, Calgary, Alberta, T2N 1N4, Canada

*****chhugenh@ucalgary.ca

**Supplementary Dataset File 1:** Flyability of 100 most populated world cities (as defined by projected population values for 2020 from the United Nations). For each flyability category, the minimum and maximum values are bolded. In this paper, we use the term “city” to refer to an “urban agglomeration”. We quantify the “impact” of these flyability restrictions by multiplying the population by number of yearly hours that are unsuitable for flying to better visualize the sum effect of flyability restrictions on people in these cities. CD is the common drone class and WRD is the weather-resistant drone class.

**Supplementary Dataset File 2:** This file contains a list of the top 500 small-drone commercial registrations in the United States as of 05 May 2020 based on data we received from the US Federal Aviation Administration. Additional sheets contain weather tolerances for the top 50 common drones and for weather-resistant drones.
